# Association between rosacea and helicobacter pylori infection: A meta-analysis

**DOI:** 10.1371/journal.pone.0301703

**Published:** 2024-04-04

**Authors:** Ying Gao, Xiao-jing Yang, Yun Zhu, Ming Yang, Fei Gu

**Affiliations:** Department of Dermatology, Children’s Hospital Capital Institute of Pediatrics, Beijing, China; Beijing University of Chinese Medicine, CHINA

## Abstract

**Background and objectives:**

The potential association between rosacea and a heightened prevalence of Helicobacter pylori (HP) infection has been previously suggested. However, existing studies offer inconsistent results. This systematic review and meta-analysis aimed to elucidate the relationship between rosacea and HP infection.

**Methods:**

We conducted comprehensive searches of PubMed, Embase, and Web of Science databases to identify relevant observational studies for our investigation. We utilized the random-effects model to aggregate the data to address the potential influence of heterogeneity among the studies on the outcome.

**Results:**

Our analysis incorporated twenty-five datasets from 23 case-control and cross-sectional studies, encompassing 51,054 rosacea patients and 4,709,074 controls without skin disease. The pooled results revealed a significantly higher prevalence of HP infection in individuals with rosacea compared to controls (odds ratio [OR]: 1.51, 95% confidence interval [CI]: 1.17–1.95, p<0.001; I^2^ = 79%). Subgroup analysis indicated an increased prevalence of HP infection in rosacea studies that utilized one (OR: 1.72, 95% CI: 1.11–2.66, p = 0.02; I^2^ = 76%) or more tests for HP infection (OR: 2.26, 95% CI: 1.29–3.98, p = 0.005; I^2^ = 56%). However, this association was not observed in population-based studies that determined HP infection based on prescription records for HP eradication drugs (OR: 0.90, 95% CI: 0.76–1.07, p = 0.024; I^2^ = 54%).

**Conclusion:**

Rosacea may be significantly associated with a higher prevalence of HP infection. High-quality prospective studies with delicately controlled confounding factors are needed to determine if HP infection is a risk factor for rosacea.

## Introduction

Rosacea is a persistent inflammatory dermatological condition distinguished by recurring facial flushing, erythema, pustules, and telangiectasia [1–3]. The cutaneous manifestations of rosacea primarily impact the central facial regions, encompassing the cheeks, nose, chin, forehead, and eyes [4]. The prevalence of rosacea exhibits considerable variation within the population, with reported figures ranging from 0.9% to 10%, contingent upon the demographic characteristics of the studied population [5]. Risk factors associated with rosacea encompass female gender, middle age, race, alcohol consumption, and excessive ultraviolet (UV) exposure [6]. The pathogenesis of rosacea remains largely unknown, with suggestions of involvement from various complex factors, including innate and adaptive immune disorder, impaired neurovascular signaling pathways, chronic inflammatory response, and overgrowth of commensal skin microorganisms [7–9]. Consequently, further efforts are required to comprehend the comorbidities and risk factors associated with the pathogenesis of rosacea.

Helicobacter pylori (HP) is a Gram-negative, spiral-shaped bacterium primarily found in the gastric mucosa of humans [10]. Traditionally, HP infection has been associated with various gastrointestinal disorders [11, 12]. However, emerging evidence indicates that HP infection also plays a role in extra-gastric diseases, including cardiovascular and neurological disorders [13]. In addition to inducing gastric mucosal inflammation, HP infection can disrupt physiological processes such as vasodilation, inflammation, and immune regulation, which have also been observed in dermatological conditions like rosacea [14, 15]. As previous research findings have yielded inconsistent results [16], this study aims to assess the potential association between rosacea and HP infection. To achieve this, a systematic review and meta-analysis were conducted.

## Materials and methods

The study adhered to the guidelines set forth by the Preferred Reporting Items for Systematic Reviews and Meta-Analyses statement [17, 18] and the Cochrane Handbook of Systematic Reviews of Interventions [18] during all stages of planning, conducting, and reporting. The meta-analysis protocol has been registered on the International Platform of Registered Systematic Review and Meta-analysis Protocols (INPLASY, https://inplasy.com/) with the registration code INPLASY2023110011.

### Inclusion and exclusion criteria of studies

The inclusion criteria were formulated based on the Population, Intervention, Comparison, Outcomes, and Study (PICOS) design guidelines, as well as aligned with the objective of the meta-analysis.

P (participants): Patients with rosacea were included as cases, and participants without skin diseases were included as controls.

I (exposure): Diagnosing rosacea was considered as exposure, consistent with the criteria used among the included studies.

C (control): Participants without skin diseases were selected as controls.

O (outcomes): The odds ratio (OR) for the prevalence or the incidence of HP infection was reported and compared between patients with rosacea or controls, or these data could be calculated. The diagnosis methods for HP infection were also consistent with those applied in the original studies.

S (study design): Observational studies, such as cohort studies, case-control studies, and cross-sectional studies.

The analysis excluded reviews, editorials, and meta-analyses. Furthermore, studies were excluded if they did not involve patients with rosacea, lacked control groups, or failed to report the outcome of HP infection. In cases where there was overlap in the populations being studied, the meta-analysis incorporated the study with the largest sample size.

### Search of databases

A comprehensive search was conducted in electronic databases, namely PubMed, Embase, and Web of Science, from their inception until August 07, 2023, to identify studies published within this timeframe. The search strategy involved combining the terms "rosacea" and "Helicobacter pylori" OR "H. pylori" OR "HP." Only full-length articles published in English and peer-reviewed journals were included in the analysis. Additionally, relevant original and review articles were manually screened for potential studies of interest.

### Data extraction and quality evaluation

Two authors independently undertook the literature searches, data collection, and study quality assessments. In the event of discrepancies, a third author was brought in to foster discussion and achieve consensus. Regarding the studies included in the analysis, we compiled exhaustive information encompassing study details, participant counts, methodologies employed for diagnosing HP infection, and the number of subjects with HP infection in both the case and control groups. Additionally, we recorded the variables that were matched or adjusted for during the analysis of the association between rosacea and HP infection.

To evaluate the quality of each study, we used the Newcastle–Ottawa Scale (NOS) (19), which assesses based on criteria such as participant selection, group comparability, and the validity of the outcomes. This scale awards up to nine stars, with a higher score indicating a more rigorous study.

### Statistics

The current study utilized the odds ratio (OR) and its corresponding 95% confidence interval (CI) to summarize the association between rosacea and HP infection. Whenever feasible, the OR and CI obtained from the regression model with the most comprehensive adjustment were extracted. In order to achieve stability and normalize variance, a logarithmic transformation was applied to the OR and its corresponding standard error (SE) in each study [18]. The Cochrane Q test and the I^2^ statistic [19] were employed to evaluate heterogeneity between studies, with an I^2^ value exceeding 50% indicating substantial heterogeneity. The results were combined using a random-effects model, which has been recommended for accounting for potential heterogeneity between studies [18]. Subgroup analyses were conducted to assess the impact of different methods for diagnosing HP infection on the findings. Publication bias was assessed using a funnel plot and Egger’s regression asymmetry test, which relied on visual symmetry judgments [20]. Statistical analyses were performed using RevMan (Version 5.1; Cochrane Collaboration, Oxford, UK) and Stata software (Version 12.0; Stata Corporation, College Station, TX, USA).

## Results

### Literature search and study retrieval

**Fig 1:**
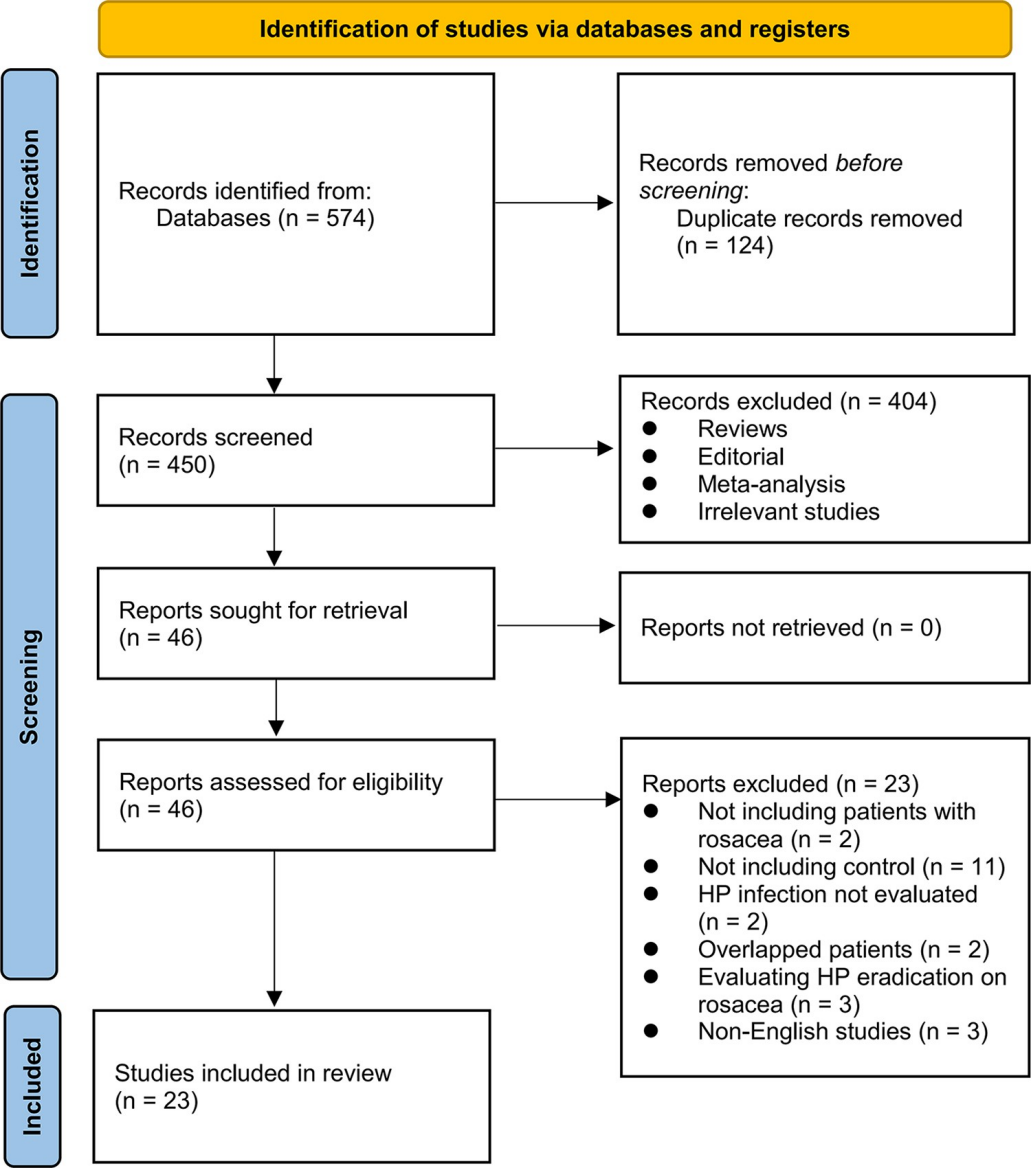
Flowchart of database search and study inclusion.

**Fig 1** depicts the sequential steps in the literature search and study retrieval process. Initially, a total of 574 records were obtained through the database search. Subsequently, 124 duplicate entries were identified and eliminated. Upon scrutinizing the titles and abstracts, 404 studies were excluded due to their lack of alignment with the meta-analysis objectives. Further examination of the full texts of the remaining 46 studies resulted in the exclusion of an additional 23 studies, with the specific rationales for exclusion outlined in **Fig 1**. Ultimately, 23 studies were chosen for inclusion in the final meta-analysis [21–43].

### Study characteristics

Overall, 23 observational studies, including 21 case-control studies [21–39, 41, 43] and two cross-sectional studies [40, 42] were included in the meta-analysis. The characteristics of the included studies are summarized in **Table 1**. These studies were published between 1998 and 2023 and were performed in the United States, Turkey, Poland, Spain, Brazil, Korea, Iran, Estonia, Greece, Egypt, Nepal, Italy, and Denmark. Overall, 51054 patients with rosacea and 4709074 controls with no skin disease were included. Most of the included studies [21–39, 41, 43] used one or more tests for the diagnosis of HP infection, which included a serological test for antibodies of HP, ^13^C or ^14^C urea breath test, HP stool antigen test, and endoscopic biopsy with histology or rapid urease test. For the other two population-based cross-sectional studies, HP infection was determined by the prescription records of drugs specific to HP eradication. Overall, 234788 participants (4.9%) were diagnosed with HP infection. Variables, including age, sex, race, and socio-economic status, were matched or adjusted to varying degrees when the association between rosacea and HP infection was reported. The NOS scores for the included studies ranged from six to nine stars, indicating that they were of moderate to good quality, respectively (**Table 2**).

**Table 1:** Characteristics of the included studies.

Study	Design	Country	No. of patients with Rosacea	No. of controls without Rosacea	Methods for evaluating HP infection	No. of participants with HP infection in cases	No. of participants with HP infection in controls	Variables matched or adjusted
Jones 1998	CC	USA	52	133	Serology for case and endoscopic biopsy for control	12	29	Age and sex
Sharma 1998	CC	USA	45	43	Serology (IgG)	12	15	Age and race
Utas 1999	CC	Turkey	25	87	Serology (IgG)	17	42	Age and sex
Dubla-Berner 1999	CC	Poland	35	28	Serology and endoscopic biopsy (histology and RUT)	25	13	Age and sex
Rojo-Garcia 2000	CC	Spain	63	567	^13^C-UBT, serology (IgG), and endoscopic biopsy	37	307	Age and sex
Bonamigo 2000	CC	Brazil	62	124	Serology (IgG)	41	72	Age, sex, and race
Herr 2000	CC	Korea	50	50	Endoscopic biopsy	42	39	Age and sex
Basak 2001	CC	Turkey	19	38	Serology (IgG)	12	23	Age and sex
Gurer 2002	CC	Turkey	33	20	Serology (IgG)	30	12	Age and sex
Szlachcic 2002	CC	Poland	60	60	^13^C-UBT, serology (IgG), and endoscopic biopsy (histology and RUT)	53	39	Age and sex
Zandi 2003	CC	Iran	29	29	Serology (IgG)	24	17	Age and sex
Baz 2004	CC	Turkey	29	20	Serology (IgG)	9	13	Age and sex
Abram 2010	CC	Estonia	85	232	Serology (IgG)	66	174	Age and sex
Lazaridou 2010	CC	Greece	100	100	Serology (IgG)	42	46	Age and sex
El-Khalawany 2012	CC	Egypt	68	54	Endoscopic biopsy (histology)	49	25	Age and sex
Bhatarai 2012	CC	Nepal	26	52	Serology (IgG)	17	6	Sex
Colgecen 2015	CC	Turkey	21	30	^14^C-UBT	16	14	Age
Gravina 2015	CC	Italy	90	90	^13^C-UBT and HpSA	44	24	Age, sex, and SES
Egeberg 2017	CS	Denmark	49475	4312213	Prescription of medications used specifically for HP	1620	102979	Age, sex, SES, smoking, alcohol abuse, and healthcare consumption
Agnoletti 2017	CC	Italy	60	40	^13^C-UBT	13	6	Age and sex
Emiroglu 2018	CC	Turkey	47	27	Serology (IgG)	25	18	Age and sex
Cho 2021	CS	Korea	520	394972	Prescription of medications used specifically for HP	135	128464	Age, sex, SES, and residence
Aghaei 2023	CC	Iran	60	65	HpSA	60	10	Age

HP, Helicobacter Pylori; CC, case-control; CS, cross-sectional; RUT, rapid urease test; HpSA, H. pylori stool antigen; UBT, Urea Breath Test; SES, socio-economic status

**Table 2:** Study quality evaluation via the Newcastle-Ottawa Scale.

Study	Adequate definition of cases	Representativeness of cases	Selection of controls	Definition of controls	Control for age and sex	Control for other confounders	Exposure ascertainment	Same methods for events ascertainment	Non-response rates	Total
Jones 1998	1	0	1	1	1	0	1	0	1	6
Sharma 1998	1	0	1	1	0	0	1	1	1	6
Utas 1999	1	0	1	1	1	0	1	1	1	7
Dubla-Berner 1999	1	0	1	1	1	0	1	1	1	7
Rojo-Garcia 2000	1	0	1	1	1	0	1	1	1	7
Bonamigo 2000	1	0	1	1	1	0	1	1	1	7
Herr 2000	1	0	1	1	1	0	1	1	1	7
Basak 2001	1	0	1	1	1	0	1	1	1	7
Gurer 2002	1	0	1	1	1	0	1	1	1	7
Szlachcic 2002	1	0	1	1	1	0	1	1	1	7
Zandi 2003	1	0	1	1	1	0	1	1	1	7
Baz 2004	1	0	1	1	1	0	1	1	1	7
Abram 2010	1	1	1	1	1	0	1	1	1	8
Lazaridou 2010	1	0	1	1	1	0	1	1	1	7
El-Khalawany 2012	1	0	1	1	1	0	1	1	1	7
Bhatarai 2012	1	0	1	1	0	0	1	1	1	6
Colgecen 2015	1	0	1	1	0	0	1	1	1	6
Gravina 2015	1	1	1	1	1	1	1	1	1	9
Egeberg 2017	1	1	1	1	1	1	0	1	1	8
Agnoletti 2017	1	0	1	1	1	0	1	1	1	7
Emiroglu 2018	1	0	1	1	1	0	1	1	1	7
Cho 2021	1	1	1	1	1	1	0	1	1	8
Aghaei 2023	1	0	1	1	0	0	1	1	1	6

### Association between rosacea and HP infection

**Fig 2:**
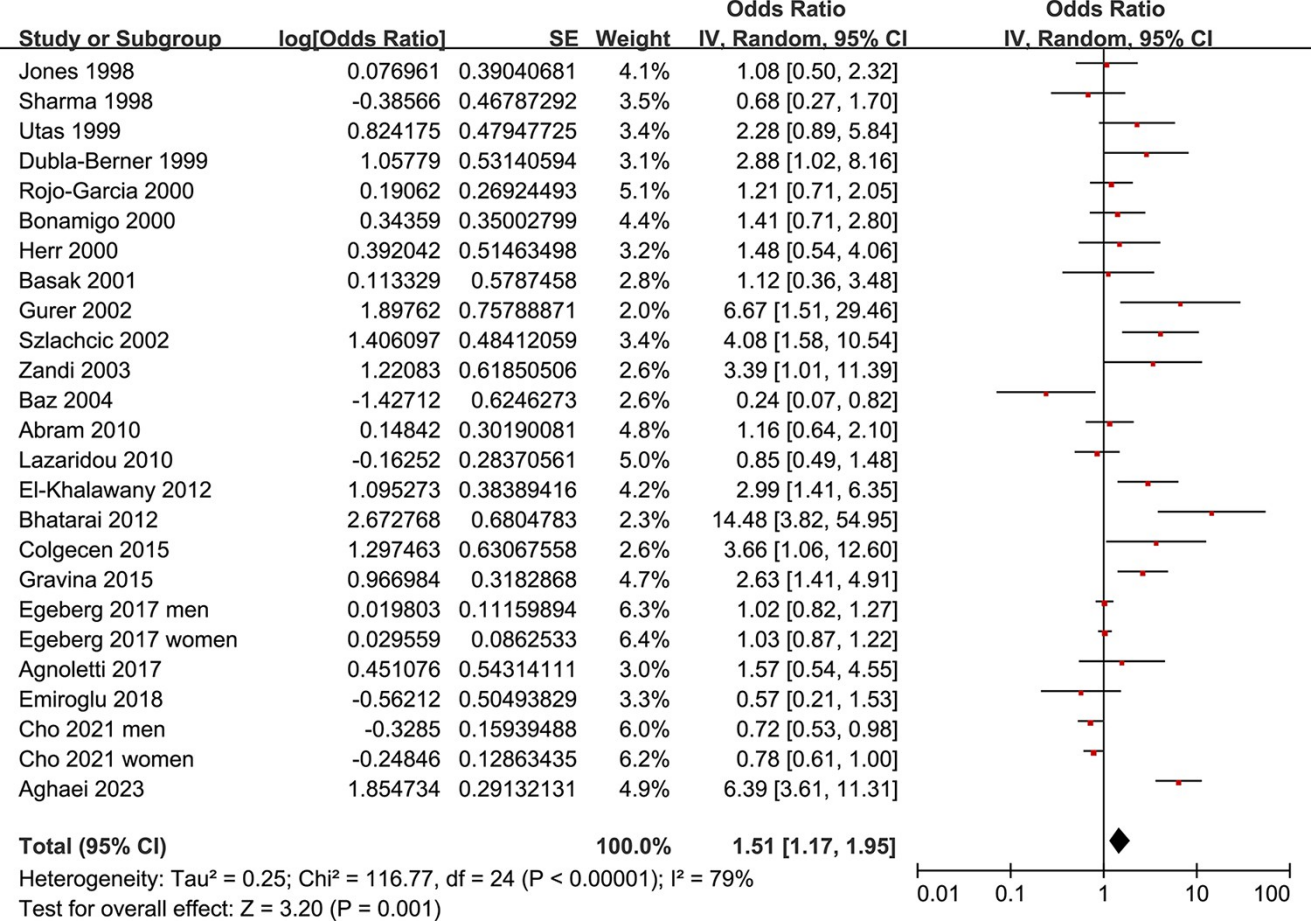
Forest plots for meta-analyses of the association between rosacea and the prevalence of HP infection.

**Fig 3:**
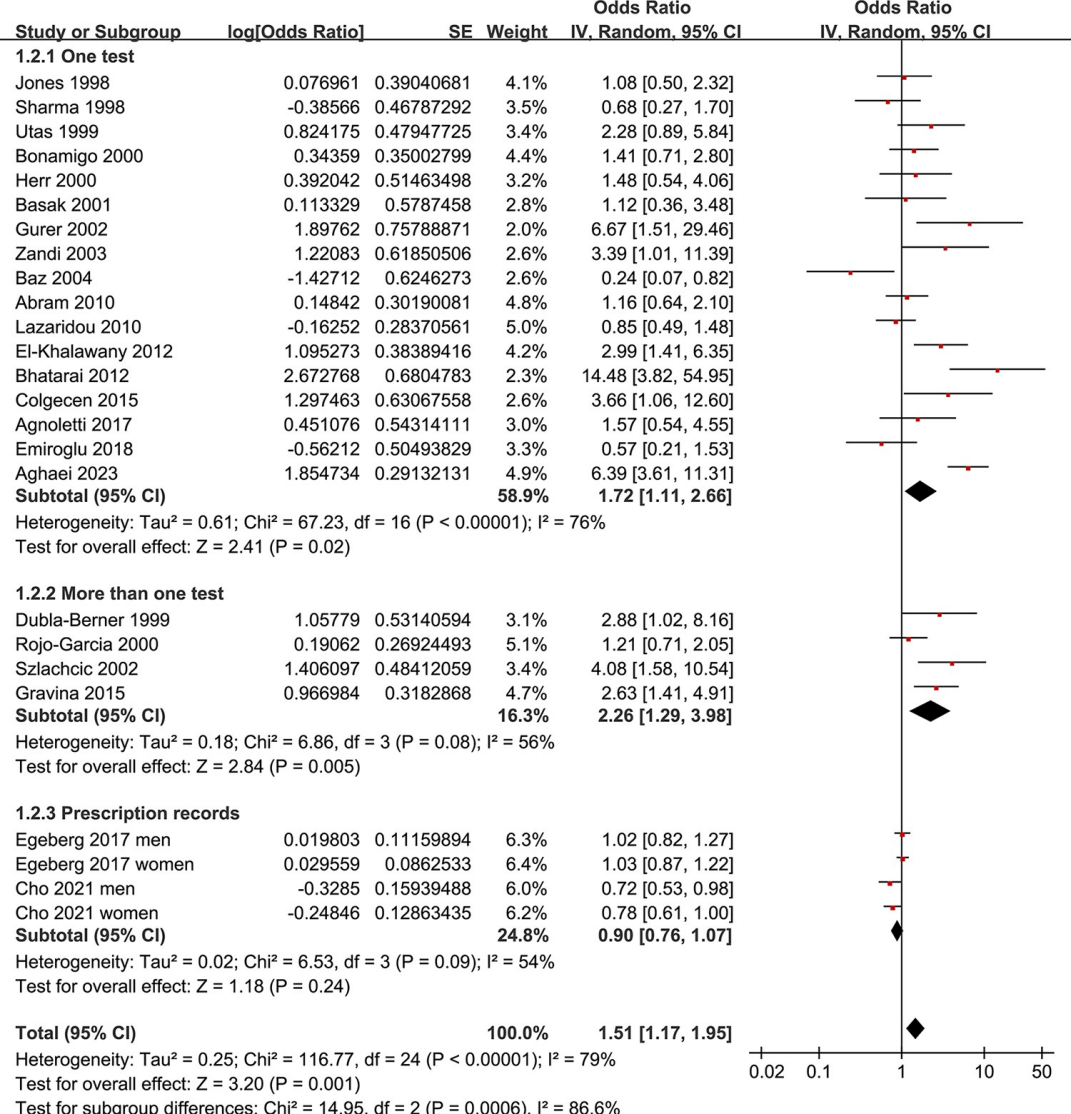
Forest plots for subgroup analysis of the association between rosacea and the prevalence of HP infection according to the strategies for determination of HP infection.

Since two studies reported two datasets in males and females separately, these datasets were included in the meta-analysis independently [40, 42]. Overall, 25 datasets from 23 studies evaluated the association between rosacea and the prevalence of HP infection. Pooled results showed that compared to controls without rosacea, patients with rosacea were associated with a higher overall prevalence of HP infection (OR: 1.51, 95% CI: 1.17 to 1.95, *p* = 0.001; **Fig 2**) with significant heterogeneity (*I*^2^ = 79%). A subsequent subgroup analysis according to the modalities for the diagnosis of HP infection suggested that rosacea was associated with a higher prevalence of HP infection in studies with one (OR: 1.72, 95% CI: 1.11 to 2.66, *p* = 0.02; *I*^2^ = 76%) or more tests for HP infection (OR: 2.26, 95% CI: 1.29 to 3.98, *p* = 0.005; *I*^2^ = 56%), but not in population-based studies with HP infection as suggested by the prescription records of HP eradication drugs (OR: 0.90, 95% CI: 0.76 to 1.07, *p* = 0.024; *I*^2^ = 54%; **Fig 3**). The difference among the subgroups was significant (*p<*0.001).

### Publication bias

**Fig 4:**
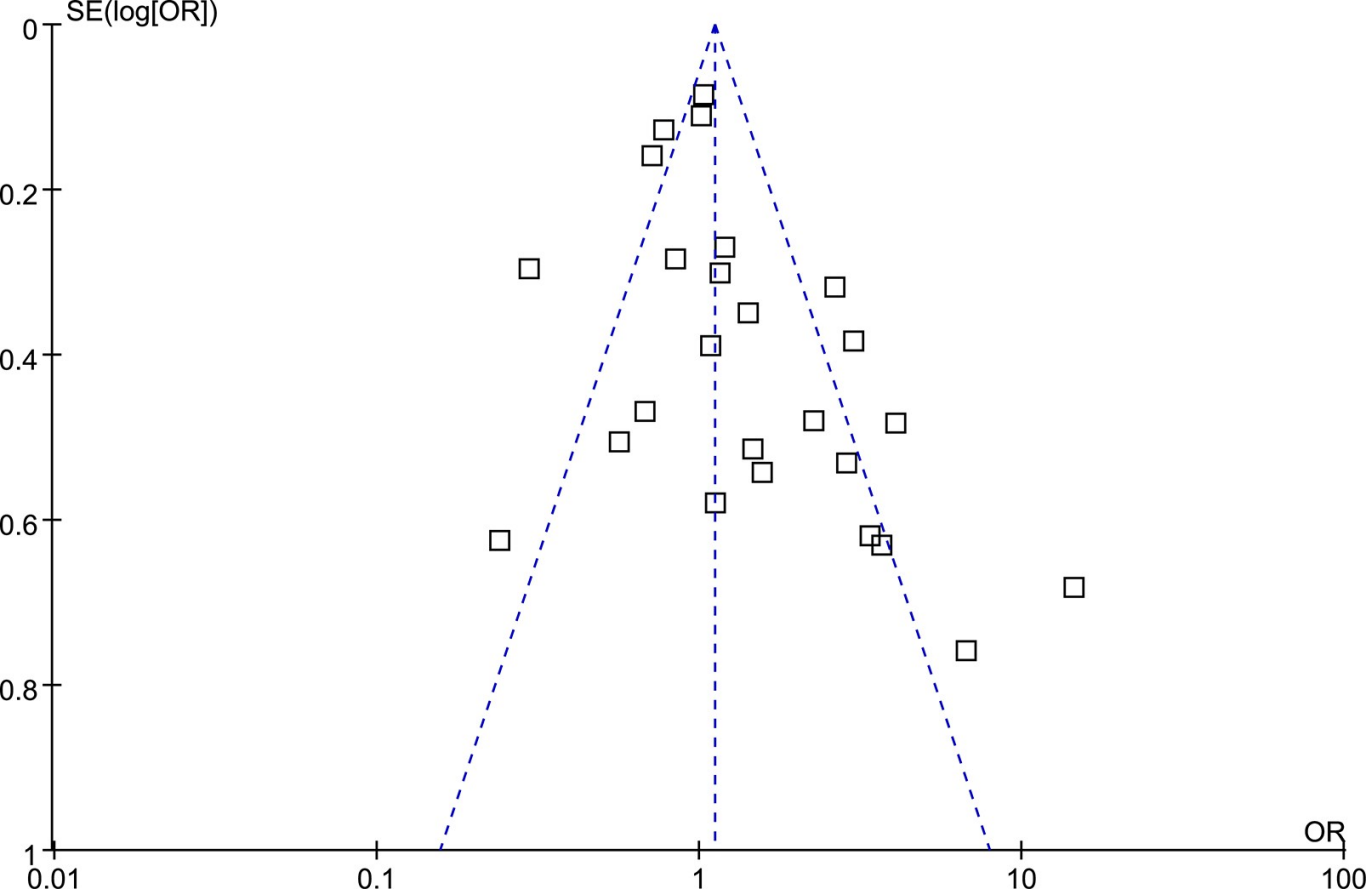
Funnel plots for the publication bias underlying the meta-analysis of the association between rosacea and the prevalence of HP infection.

The funnel plots depicting the meta-analysis of the relationship between rosacea and the prevalence of HP infection are displayed in **Fig 4**. Upon visual inspection, the plots exhibit symmetry, indicating minimal publication bias. Furthermore, Egger’s regression tests yielded a low probability of publication bias (*p* = 0.41).

## Discussion

This systematic review and meta-analysis synthesized the findings from 23 observational studies, demonstrating a potential association between a higher prevalence of HP infection and rosacea compared to controls without skin diseases. Furthermore, the subgroup analysis indicated that the method of diagnosing the infection could significantly influence the meta-analysis results. Notably, a higher prevalence of HP was observed in rosacea patients in studies where HP was diagnosed through one or more clinical tests, in contrast to studies relying on prescription records for HP eradication medications to estimate HP prevalence. In summary, the meta-analysis supports a plausible correlation between rosacea and HP infection, suggesting that HP infection might play a role in the pathogenesis of rosacea.

Based on current knowledge, limited meta-analyses observed the relationship between rosacea and HP infection. An early meta-analysis in 2017 included 14 observational studies with 928 rosacea patients and 1527 controls failing to show a significant association between rosacea and HP infection [44]. Moreover, exploring meta-analysis, including seven pilot studies, also failed to show a significantly improved symptom of rosacea following HP eradication [44]. Compared to the previous meta-analysis [44], ours has multiple strengths. Initially, a comprehensive review of the existing literature was conducted across three widely used electronic databases, yielding 23 recent studies that substantiated the correlation between rosacea and HP infection. The present meta-analysis encompasses a significantly larger sample size than its predecessor [44]. Only studies published as complete articles in peer-reviewed journals were incorporated, while grey literature, such as conference abstracts, was excluded. This exclusion was motivated by the potential impact on the reliability of findings, as grey literature typically lacks the scrutiny of peer review. Moreover, potential confounding factors such as age, sex, race, and socio-economic factors were adjusted to vary among all the included studies, thereby minimizing the influence of the above factors. Finally, a subgroup analysis showed that the association between rosacea and HP infection may be significantly affected by the different strategies used to diagnose HP, which may at least partly contribute to the heterogeneity.

No consensus has been reached regarding the optimal diagnostic test for HP infection. Each frequently used test has its advantages and disadvantages [45]. Accordingly, multiple clinical tests may be more effective than applying only one test for HP detection [45]. Our subgroup analysis suggested that the association between rosacea and HP infection was stronger in studies with more than one clinical test for HP infection compared to studies with only one clinical test for HP infection (OR: 2.26 versus 1.72). However, the association became non-significant in studies with HP infection estimated by the prescription records of medications specifically for HP eradication (OR: 0.90). Although two of the studies based on the prescription records are of the largest sample size, the prevalence of HP was likely to be underestimated because a large proportion of patients with HP infection are asymptomatic and less likely to have HP eradication therapy. Therefore, the results of the subgroup with studies based on the prescription records should be interpreted with caution.

Establishing the potential association between rosacea and HP infection at the patient level is an early investigation stage with limited clinical significance. However, because the mechanisms of rosacea are not fully understood, the treatment methods are limited, and the recurrence rate is high [46], it is essential to reveal associated factors of the disease, particularly at the patient level. Clarifying the factors related to the onset of rosacea may be beneficial for subsequent intervention research, such as to determine if HP infection may be an independent risk factor of rosacea severity and whether eradicating HP infection might be beneficial to improve rosacea symptoms and reduce recurrence rate. The results of our meta-analysis are consistent with previous studies, which showed an association between HP infection and the severity of rosacea. An early study by Diaz et al. in 2003 suggested that HP infection was higher in patients with inflammatory/papulopustular rosacea than those with erythematotelangiectatic rosacea [47]. A later study including 36 patients with rosacea in Libya also showed that patients with serum IgG to HP was significantly higher in severe rosacea than moderate or mild rosacea regardless of the type of the disease [32]. Moreover, if a potential association between rosacea and HP infection could be derived, the next important question in clinical practice is determining the influence of HP eradication therapy on rosacea symptoms. Although several case reports and observational studies have suggested the benefits of HP eradication therapy on symptoms of patients with rosacea and HP infection [48, 49], an early double-blind clinical trial of 44 patients with rosacea failed to show any benefit of HP eradication for 14 days on symptoms of rosacea [50]. However, this study had a limited sample size and short follow-up duration [50], and large-scale clinical trials may be considered in the future.

This study is subject to certain limitations. Firstly, it should be noted that all the studies included in the analysis were case-control and cross-sectional studies, which prevents the establishment of a causative relationship between HP infection and rosacea. Secondly, the potential influence of uncontrolled factors, including smoking, some particular behavioral/lifestyle habits, concurrent medications and recent antibiotic use, on the association between HP infection and rosacea could not be completely ruled out. As mentioned previously, HP infection has also been involved in multiple gastric and extra-gastric diseases, including cardiovascular and neurological disorders [13]. However, the prevalence of these comorbidities was not reported or controlled between patients with and without rosacea among the included studies, which may also confound the association. High-quality prospective studies with delicately controlled confounding factors are still needed to determine if HP infection is an independent risk factor of rosacea. Additionally, it is important to acknowledge that the meta-analysis relied on aggregated data at the study level rather than individual-participant data. Hence, our study could not ascertain the impact of participant characteristics, including age, sex, race, and level of UV exposure. Finally, the subgroup analysis results should be interpreted cautiously because the heterogeneity remains significant within each subgroup of the HP detection strategy.

In conclusion, the meta-analysis reveals that individuals with rosacea exhibit a higher prevalence of HP infection than controls without skin ailments. This seems considerably influenced by the varied strategies employed in diagnosing HP infection. Given that a possible link has been established between HP infection and rosacea severity, high-quality prospective studies with delicately controlled confounding factors are needed in the future to determine if HP infection is a risk factor for rosacea.

## Supporting information

S1 Checklist PRISMA 2009 checklist. (DOC)
